# In and out among the Hospitals

**Published:** 1888-11-24

**Authors:** 


### In and Out Among the Hospitals.

By a Secretary of Ten Years' Standing.

### WEST CORNWALL MINERS' HOSPITAL, REDRUTH.

This hospital is in great part supported by Lord Robartes, who last year paid £528 7*s.* 11*d.* out of the total cost of maintenance, which amounted to £1,329 16*s.* Lord Robartes has also provided additional wards and a bathroom for the hospital at a cost of £309 18*s.* 1*d.* The number of patients treated during the year was 228, and their average stay was 40⅝ days. This is unusually long, nor does the nature of the cases treated fully account for it. No mention is made of out-patients being treated by the hospital. The average cost of food per head was 9*s.* 5*d.* per week, a sum the moderation of which is largely due to the good management of the matron, Miss Emily Angove, who received a deserved acknowledgment from the committee for her economy. It is satisfactory to note that the contributions from workmen are yearly growing larger, and that several of the subscriptions from mines are increased.

### ADDENBROOKE'S HOSPITAL, CAMBRIDGE.

This hospital, which sends so many students to the London schools of medicine after they have passed the first part of their curriculum at Cambridge, is in a flourishing condition. Last year the governors were enabled to invest £1,300 in the public funds, after defraying all expenses. The total receipts were £7,285, which includes legacies amounting to £1,200 14*s.*, and the expenditure was £5,670. Deducting 1*s.* per out-patient and the extraordinary expenditure, the cost per bed occupied was £50 10*s.* 3*d.*, and there was a daily average of 108 patients in the wards. The total number of in-patients was 915, and out-patients 3,838. The accounts as set forth in the report are not very clear, and the form in which they are presented might be remodelled and extended with much advantage to the best interests of the charity.

### SOUTH INFIRMARY, CORK.

Most hospitals and infirmaries in Ireland are voted a certain sum of money towards their support at the Presentment Sessions, and this hospital altogether receives about £1,400 per annum in this way, half of which is voted by the city and half by the county; the other sources of income are ordinary annual subscriptions and donations, and there are a few investments, besides which there was a sum of £58 15*s.*, received last year from paying patients. The total income was £2,403 15*s.* 1*d.*, and the expenditure £2,466 4*s.* 7*d.* There were altogether 1,020 patients treated in the wards, and the daily average number of beds occupied was 66; deducting 1*s.* for each out-patient, of which there were 10,642, the cost per bed occupied was £29 6*s.* 1*d.* The pay wards are quite a feature of this hospital, and it is to be wondered at that they are not more availed of by the inhabitants of Cork. Every convenience is afforded for those who are unwilling to accept the advantages of the ordinaay ward—strict privacy, careful nursing, medical and surgical treatment, food, etc., for a payment of £1 per week. A Cork man is nothing if he has not sentiment and the feelings of a poet, and one such must have compiled the last published report. There are quotations, from Carlyle down to the most humble local poet, putting forcibly before the readers the great good done by hospitals in general and the Cork South Charitable Infirmary in particular.

